# Effects of a transient lack of dietary mineral phosphorus on renal gene expression and plasma metabolites in two high-yielding laying hen strains

**DOI:** 10.1186/s12864-025-11294-6

**Published:** 2025-02-10

**Authors:** Hiba Qasir, Henry Reyer, Michael Oster, Siriluck Ponsuksili, Nares Trakooljul, Vera Sommerfeld, Markus Rodehutscord, Klaus Wimmers

**Affiliations:** 1 Research Institute for Farm Animal Biology (FBN), Wilhelm-Stahl-Allee 2, 18196 Dummerstorf, Germany; 2 Institute of Animal Science, University of Hohenheim, Emil-Wolff-Str. 10, 70599 Stuttgart, Germany; 3 Faculty of Agricultural and Environmental Sciences, University Rostock, Justus-von-Liebig-Weg 6, 18059 Rostock, Germany

**Keywords:** Kidney, Laying period, Mineral homeostasis, Poultry, Transcriptomics

## Abstract

**Background:**

There is an emerging body of evidence that current poultry feed is formulated in excess for phosphorus (P), which results in unnecessarily high P excretions. Sustainable concepts for agricultural P flows should trigger animal-intrinsic mechanisms for efficient P utilization. In the current study, Lohmann Brown (LB) and Lohmann Selected Leghorn (LSL) laying hens were fed either a high P diet (P+) with 1 g/kg mineral P supplement or a low P diet (P-) with 0 g/kg mineral P supplement for a period of 4 weeks prior to sampling. Before and after onset of laying, i.e., at 19 and 24 weeks of life, kidney and plasma samples were collected to investigate the endogenous P utilization in response to restricted dietary P, laying hen strain, and sexual maturation.

**Results:**

Plasma analyses of minerals and metabolites confirmed the response to a low P diet, which was characterized by a significant reduction in plasma P levels at week 19 in both strains. The plasma calcium (Ca) levels were tightly regulated throughout the entire experimental period. Notably, there was a numerical trend of increased plasma calcitriol levels in P- fed birds of both strains compared to the P + group, which might have mediated a substantial role regarding the adaptive responses to low P supply. At week 19, RNA sequencing of kidney identified 1,114 and 556 differentially expressed genes (DEGs) unique to the LB and LSL strains, respectively. The number of DEGs declined with increasing maturity of the hens culminating in 90 and 146 DEGs for LB and LSL strains at week 24. Analyses revealed an enrichment of pathways related to energy metabolism and cell cycle, particularly at week 19 in both strains. The diet-specific expression of target genes involved in P homeostasis highlighted transcripts related to active (*SLC34A1*, *SLC20A2*) and passive mineral transport (*CLDN14*, *CLDN16*), Ca utilization (*STC1*, *CALB1*), and acid-base balance (*CA2*, *SLC4A1*).

**Conclusions:**

Results suggest that both laying hen strains adapted to the lack of mineral P supplements and achieved a physiological Ca: P-ratio in body compartments through endogenous regulation as evidenced via the endocrine profile.

**Supplementary Information:**

The online version contains supplementary material available at 10.1186/s12864-025-11294-6.

## Background

Egg production has reached an industrial scale through the use of high-performance laying hen breeds such as Lohmann Brown (LB) and Lohmann Selected Leghorn (LSL). Dietary mineral management, particularly of phosphorus (P), is crucial for performance and animal health in these laying hens [1]. P is biologically available to laying hens in the form of non-phytate P, whereas about 60% of plant P in the form of phytate is not available for direct enteral absorption because of insufficient intestinal phytase activity [1, 2]. Therefore, the use of mineral P sources and phytases of microbial origin is very common to compensate for the low rate of digestible P obtained from cereals and other plant-based feed. Current dietary recommendations in laying hens suggest 2.2 g/kg non-phytate P (NPP) [3], however, these levels exceed the required amount for achieving optimal performance and jeopardize efforts to reduce the environmental P emissions [4]. Current approaches have monitored experimental hen populations for intestinal phytate cleavage and resulting *myo*-inositol and inositol phosphate isomer levels in the small intestine following two variable P diets (5.3 vs. 4.7 g/kg total P) and suggested that a 20% reduction in currently recommended P levels could be achieved in commercial laying hens [5]. A feasible potential to reduce dietary mineral P levels might still be larger, as a reduction in available P from 491 to 168 mg/hen/d in hens’ feed did not affect the egg production and health parameters but lowered the amount of excreted P in laying hens [1].

The adaptability of poultry to variations in dietary P levels has been attributed to their body P homeostasis network [6], maintained through a complex interaction of multiple tissues (intestines, kidney, liver, bone), active and passive mineral transport, as well as feedback mechanisms due to endocrine regulators [7–9]. These findings were evident in broiler chickens and laying hens, where recent reports on low-P diets found regulatory changes at the level of the endocrine system and gene expression in the small intestine [5, 10, 11] and kidney [12]. Sexual maturation (onset of laying), commencing around the 19th week of life and extending beyond the 24th week, is characterized by an increased mineral demand in laying hens due to eggshell production, which links the P metabolism particularly closely with the calcium (Ca) metabolism [13, 14]. In this context, besides intestinal tissues, the contribution of post-absorptive tissues (i.e. kidney) to mineral utilization is of relevance.

The renal mineral transport includes para- and transcellular processes, e.g. facilitated by *SLC34A1*, which encodes the type 2a sodium-phosphate cotransporter (NPt2a) in chickens [12, 15], components of tight junctions such as claudin 14 (*CLDN14*), and intracellular carriers such as calbindin (*CALB1*). Approximately 80% of the absorption of P occurs in the proximal renal tubules through apically localized Na-dependent P cotransporters [16]. Moreover, proximal tubular Na/P cotransport is enhanced by stanniocalcin 1 (*STC1*) [17]. In mammals, parathyroid hormone (PTH) regulates Ca homeostasis by altering the expression of the renal Ca channel TRPV5 in the distal convoluted tubule and collecting duct [18]. In addition, it causes a decreased abundance of renal NPt2a in the apical membrane of the proximal tubule [16]. Increased blood Ca levels suppress the synthesis and secretion of PTH from the parathyroid glands by binding to and activating the Ca-sensing receptor (CaSR). CaSR antagonizes PTH effects in proximal cells by inhibiting cAMP production and promotes P reabsorption by NaPt2 in the proximal tubule. Furthermore, PTH acts on renal 1-α hydroxylase to form calcitriol (active form of vitamin D3; 1,25-(OH)2D3) from calcidiol (storage form of vitamin D3; 25(OH)D3) in the kidney [19]. These interactive mechanisms modulate bone resorption, Ca and P absorption in the intestine and reabsorption in the kidney to maintain physiological Ca and P concentrations in the circulation [8, 20].

High-performance laying hen strains of LB and LSL genotypes are characterized by a considerable genetic differentiation [21, 22], which culminates in distinct differences in phenotypic traits such as body weight and egg quality traits, including egg weight and eggshell weight. In fact, the underlying cause for the observed variations can be attributed to divergent breeding histories [23]. Subsequent breeding programs for both LB and LSL strains primarily focused on traits such as feed efficiency and laying performance, resulting in a similarly high egg production capacity of the two strains. According to recent findings, LB and LSL exhibit significantly differences in endogenous responses towards improved mineral utilization via enhanced intestinal capacity [5], jejunal microbial composition [24], jejunal gene expression [25], immune traits [26], and endocrine drivers [11]. Therefore, we hypothesize that high-yielding laying hens, when fed a low P diet at the onset of egg production, initiate endogenous adaptive mechanisms in post-absorptive tissues such as the kidneys to maintain physiological mineral equilibrium. To address this, the effects of a low P diet induced by a 4-week removal of mineral P sources from the diet compared to a diet containing mineral P on renal gene expression of LB and LSL laying hens before (week 19) and after the onset of laying (week 24) were investigated. Furthermore, the study examined the impact of diet, strain, and maturation on plasma metabolites relevant to mineral utilization.

## Materials and methods

### Ethics and consent to participate

This study was part of the interdisciplinary Research Unit P-Fowl: Inositol phosphates and myo-inositol in the domestic fowl: Exploring the interface of genetics, physiology, microbiome, and nutrition (https://p-fowl.uni-hohenheim.de/). Newly hatched female chickens were retrieved from a breeding company (Lohmann Breeders GmbH, Cuxhaven, Germany). The informed consent of the owner(s) for the use of the animals in the study has been provided. The animal trial was performed at the Agricultural Experimental Station of the University of Hohenheim, Germany. The experimental protocol was in strict compliance with the German Animal Welfare Legislation and approved by regional Ethics committee, i.e., the Regierungspräsidium Tubingen, Germany, and the Animal Welfare Committee of the University of Hohenheim (Project no. HOH67-21TE).

### Experimental setup and laying hens

As reported previously, both LB (*n* = 40) and LSL (*n* = 40) hens were supplied with experimental maize-soybean meal-based diets having all required nutrients in recommended doses, except P [27]. The high P diet (P+) was supplemented with 1 g P/kg. The total P concentration in the P + diet was 4.9 g/kg dry matter (DM) for developer feed, 5.0 g/kg DM for pre-layer feed, and 4.9 g/kg DM for layer feed. The experimental low P diet (P-) lacked any mineral P supplement and the respective total P content was 3.7 g/kg DM for developer feed, 3.8 g/kg DM for pre-layer feed, and 3.6 g/kg DM for layer feed. The respective non-phytate P (NPP) values in the P-supplemented group were calculated as 2.3 g/kg (developer feed), 2.4 g/kg (pre-laying feed), and 2.3 g/kg (layer feed) to match current NPP recommendations [27, 28]. All diets lacked phytase supplements. Animals sampled at week 19 received developer feed (week 15–16), pre-layer feed (week 16–17), and layer feed (week 17–19) according to their affiliated dietary group; animals sampled at week 24 received respective layer diets from week 20–24 (Table S1). The hens were fed the experimental diets for a period of four weeks before slaughtering in week 19 (*n* = 40) and week 24 (*n* = 40), respectively, where kidney (*n* = 80) and plasma samples (*n* = 80) were collected, i.e., 10 hens per strain per sampling week per diet. The feed composition remained consistent at both sampling stages to ensure that all sampled birds received layer feed. Hens were killed from 09:00 h to 15:00 h through individual stunning with a gas mixture (35% CO_2_, 35% N_2_, 30% O_2_) and decapitation. Trunk blood was collected in lithium-heparin tubes, centrifuged, and the prepared plasma samples were stored at − 80 °C until analyses. Whole kidneys were collected approximately 5 min postmortem. The tissue was chopped into small pieces and frozen at − 80 °C until analyses. The number of eggs laid until slaughter was documented for all animals. Until week 24, the average number of eggs produced were 10.0 ± 5.8 for LB and 12.0 ± 3.5 for LSL. A total of 3 laying hens from the LB strain affiliated with both P- and P + diet groups showed no egg production until week 24 (i.e. delayed sexual maturation or infertility) and were excluded from the subsequent plasma analyses.

### Measurement of plasma parameters

The plasma samples were analyzed for inorganic P, total Ca, magnesium (Mg), albumin, triacylglycerides, and alkaline phosphatase activity (ALP) using the Fuji DriChem 4000i commercial assays (FujiFilm, Minato, Japan). Commercially available enzyme-linked immunosorbent assays (ELISA) were used to quantify hormones in duplicate according to the manufacturer’s instructions. The measured hormones were 25(OH) vitamin D (EIA-5396, DRG, Marburg, Germany), 1,25(OH) vitamin D (AC-62F1, Immunodiagnostic Systems GmbH, Frankfurt am Main, Germany), and PTH (CSBE118880Ch, CusaBio, Houston, USA). The raw data were processed according to 4-parameter logistic curve analysis with MARS data analysis software (BMG Labtech, Ortenberg, Germany). For data analysis, a linear model was applied including sampling week, diet group, strain, and interactions as fixed effects (R, version 4.3.0, nmle package v3.1-160). The statistical model was structured as follows:

y_ijk_ = µ + strain_i_ + week_j_ + diet_k_ + (strain × week × diet)_ijk_ + ϵ_ijk_.

Where y_ijk_ is the response variable, µ is the overall mean, strain_i_ represents the effect of genotype (LB, LSL), week_j_ represents the effect of week (wk19, wk24), and diet_k_ represents the effect of dietary P level (P-, P+), and ϵ_ijk_ is the residual error. The ANOVA and TukeyHSD functions were applied to analyze the differences among means between different experimental groups. The significance cut-off was set at a *P*-value < 0.05. The results were displayed in graphical form using R (ggplot2 package v3.3.6).

### RNA extraction and processing of sequencing data

Before RNA extraction, whole frozen kidney samples were ground with pestle and mortar while submerged in liquid nitrogen. About forty grams of each kidney sample were used for RNA isolation using TRI reagent (Sigma-Aldrich, Taufkirchen, Germany) and phenol-chloroform-based RNA extraction according to the TRI reagent protocol. To ensure the absence of DNA, the isolated RNA was treated with DNase I (Roche Diagnostics, Mannheim, Germany) and finally purified using the NucleoSpin RNA extraction kit (Macherey-Nagel, Düren, Germany). The RNA concentration was determined through spectrophotometry using the NanoDrop ND-2000 (Peqlab, Erlangen, Germany) and RNA quality of final RNA was analyzed with the Bioanalyzer 2100 device (Agilent Technologies, Waldbronn, Germany). The range of RNA integrity numbers (RIN) was between 6.4 and 8.9, with an average of 7.8. The absence of genomic DNA contamination was confirmed with polymerase chain reaction (PCR) specific for a chicken GAPDH fragment [12]. RNA libraries were prepared using the Illumina Stranded mRNA preparation kit (Illumina, San Diego, CA, USA) and then sequenced using the Illumina NextSeq 2000 platform in a paired-end configuration. Quality control and preprocessing of the raw sequencing reads were conducted using FastQC (v0.11.7) and Trim Galore (v0.5.0; https://www.bioinformatics.babraham.ac.uk/projects/). Low-quality reads with mean Q-score less than 20 and reads length shorter than 30 base pairs were excluded. The remaining reads were aligned to the chicken genome assembly (GRCg6a, Ensembl release 109) using Hisat2 (v2.1.0; http://daehwankimlab.github.io/hisat2/). HTseq (v0.11.2) was used to summarize read counts to determine the expression levels of each gene. Data are available from the EMBL-EBI database (www.ebi.ac.uk/arrayexpress) via the accession number E-MTAB-14,436. Each of the 80 kidney samples yielded an average of 22.06 ± 6.80 million paired end reads for downstream analysis. Following analyzing the dataset using the arrayQualityMetrics package (R, v3.54), 75 samples were used with 5 outliers removed (2 birds from LB P + group at week 19, 1 from LSL P + at week 19, and 2 from LSL P- group at week 19). In the project consortia, a number of candidate genes from an RNAseq run were validated previously on a LightCycler 480 device (Roche, Basel, Switzerland), demonstrating high consistency between RNAseq data and qRT-PCR results [29].

### Gene expression analysis

The count data were passed through strict filtration with 10 or more counts in at least 8 samples for subsets representing strain × stage. The gene expression analysis was performed with the DESeq2 package in R (v1.38.3) per strain and stage to elucidate dietary effects using the Wald test. A size factor normalization was applied to account for sequencing depth differences between samples. The statistical model comprised strain (LB, LSL), week (wk19, wk24), and diet group (P-, P+) as fixed effects. Analysis of the applied P diets considered the following four contrasts, whose labels were derived from strain and week and diet: (i) LBwk19P+ (*n* = 8) vs. LBwk19P- (*n* = 10), (ii) LSLwk19P+ (*n* = 9) vs. LSLwk19P- (*n* = 8), (iii) LBwk24P+ (*n* = 10) vs. LBwk24P- (*n* = 10), and (iv) LSLwk24P+ (*n* = 10) vs. LSLwk24P- (*n* = 10). For each of the contrasts, differentially expressed genes (DEGs) at *P*-value < 0.01 were considered for further analyses. Venn diagrams were created for each applied contrast to display the DEGs using the online tool Venny (https://bioinfogp.cnb.csic.es/tools/venny/).

### Functional annotation and pathway enrichment analysis

The chicken Ensembl-IDs were converted to gene symbols using the online tool bioDBnet: db2db (https://biodbnet-abcc.ncifcrf.gov/db/db2db.php). For data enrichment analysis, DEGs with *P* < 0.01 were selected and analyzed through the online tool g: profiler (https://biit.cs.ut.ee/gprofiler/gost). Gene Ontology (GO: BP) and Kyoto Encyclopedia of Genes and Genomes (KEGG) were used considering maximal term sizes of 500. The KEGG pathway map was visualized using the R package ggplot2 (v3.3.6). DEGs were presented by volcano plots using VolcaNoseR (https://huygens.science.uva.nl/VolcaNoseR). Genes with a -log_10_ transformed *P-*value above 2 were considered as functional targets to drive renal adaptation due to reduced dietary P intake. In addition, a target genes approach was used to identify transcripts involved in P, Ca, *myo*-inositol, and vitamin D regulation. In total, 50 target genes from previous studies (mineral transport [15, 30–33]; vitamin D metabolism [34]; *myo*-inositol transporters [35]) were selected and compared with DEGs from the retrieved expression data.

## Results

### Plasma hormones and metabolites

**Fig. 1:**
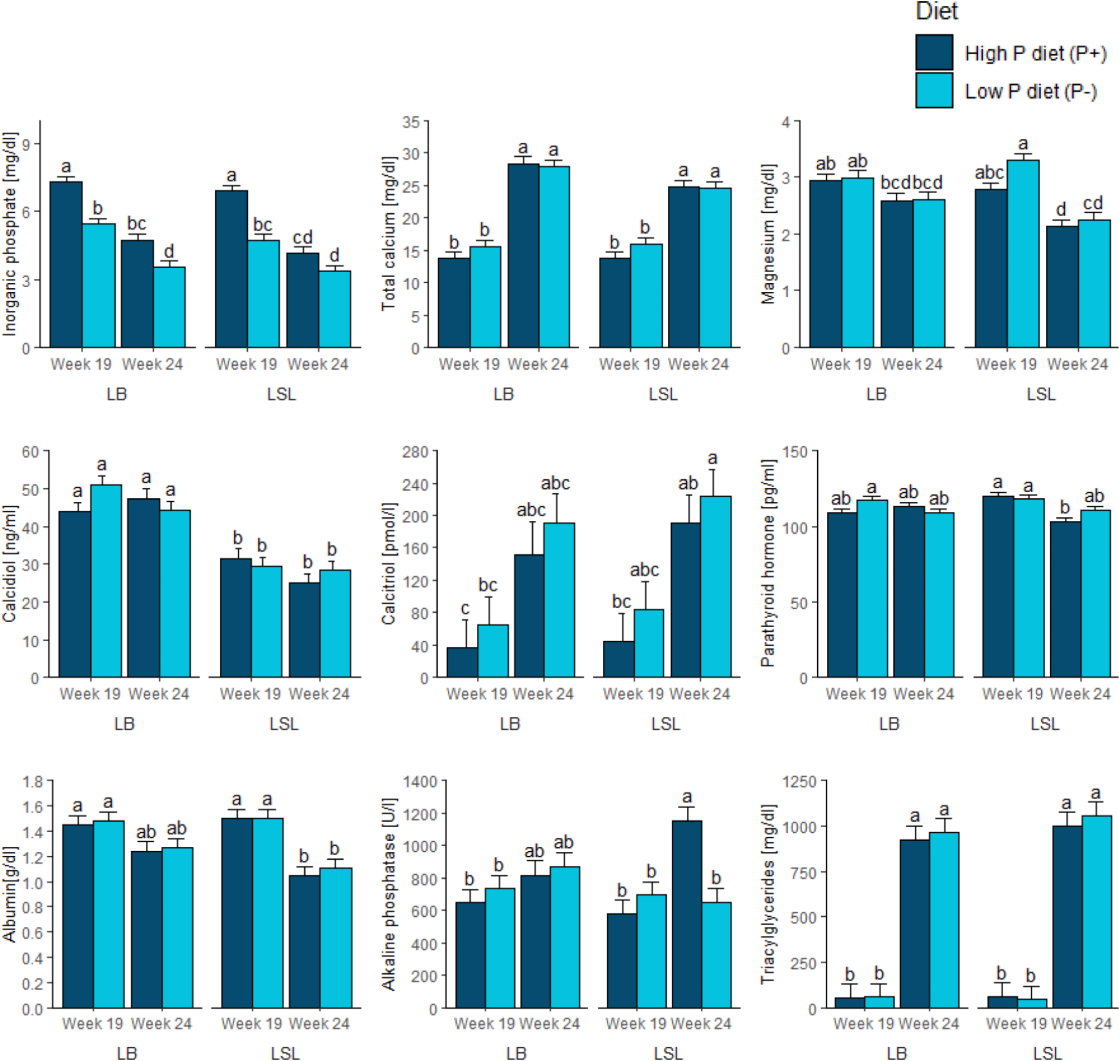
Plasma parameters related to mineral homeostasis in LB and LSL hen strains before (week 19) and after (week 24) onset of egg laying following a high P diet supplemented with 1 g/kg mineral P (P+; dark blue) or a low P diet lacking any mineral P supplements (P-, light blue). Values are presented as mean ± SE. ^a, b,c, d^ Different superscripts indicate statistical differences between the eight experimental groups, i.e., 2 strains × 2 diets × 2 stages (*P* < 0.05)

For dietary effects, hens fed P- diets exhibited a lower plasma inorganic P concentration in both strains at week 19, whereas at week 24, only LB hens showed significantly decreased inorganic P plasma levels in response to the P- diet (Fig. 1). The ALP activity was significantly increased in hens fed the P + diet compared to hens fed the P- diet at week 24 in LSL strain. No significant differences in ALP activity were observed in the LB strain at both sampling stages. Dietary P supply did not yield any significant effects on the plasma concentrations of total Ca, Mg, calcidiol, calcitriol, albumin and triacylglycerides for both LSL and LB strains at weeks 19 and 24. However, plasma calcitriol values showed a consistent pattern according to the dietary P supply with numerically increased values in P- animals of both laying hen strains.

A strain effect was observed on plasma calcidiol levels with significantly lower concentrations in the LSL strain compared to the LB strain at both weeks 19 and 24, irrespective of dietary P supply. Other analyzed metabolites did not consistently differ between the strains.

Regarding effects of the sampling stage, i.e. sexual maturation, inorganic P levels were significantly lower, while total Ca and triacylglycerides were higher at week 24 compared to week 19 for both strains and diets. Levels of plasma Mg and albumin were higher at week 19 compared to week 24 in both P + and P- diet groups in LSL but not in LB laying hens. Plasma PTH levels were higher in week 19 compared to week 24 in LSL hens fed the control P + diet. In contrast, ALP activity levels were higher in week 24 compared to week 19 only in LSL hens within the control group (P + diet). No consistent effects of sampling stage were observed on plasma concentrations of calcidiol, calcitriol, and PTH. Even though plasma calcitriol showed no significant differences, there were consistent patterns among all hen groups with numerically higher calcitriol levels at week 24 compared to week 19. The statistical evaluation of the fixed effects considered in the linear model showed that the dietary P supply significantly influenced plasma concentrations of inorganic P (*P* < 0.001) and PTH (*P* = 0.035). Strain exhibited a significant effect on plasma calcidiol (*P* < 0.001) and PTH (*P* = 0.005), whereas sampling stage showed a significant effect on plasma inorganic P (*P* < 0.001), total Ca (*P* < 0.001), calcitriol (*P* = 0.035) and triacylglycerides (*P* < 0.001). Interaction effects of diet × strain × sampling stage were observed for plasma calcidiol (*P* = 0.033), PTH (*P* = 0.007), and ALP activity (*P* = 0.019).

### Differential gene expression analysis

**Fig. 2:**
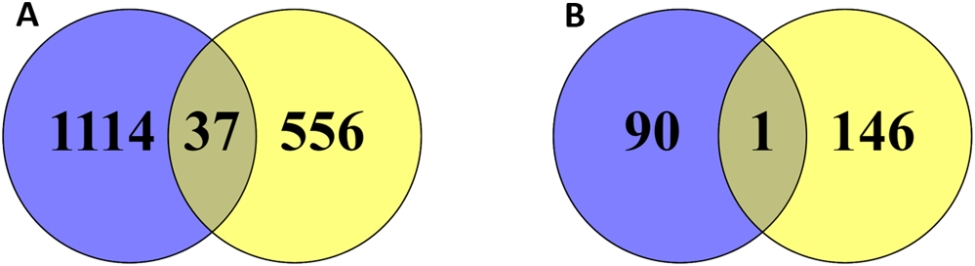
Venn diagrams of differentially expressed genes (DEGs) in LB (blue) and LSL (yellow) hens under low P diet (P-) compared to a high P diet (P+) at week 19 (**A**) and week 24 (**B**)

The gene expression analysis in the kidney of LB and LSL laying hen strains at week 19 and 24 kept on different dietary P supply (P + vs. P-) revealed 1,114 and 556 DEGs specific to the LB strain and the LSL strain, respectively, at week 19 (Fig. 2A). At week 24, 90 DEGs were observed specifically in LB and 146 DEGs specifically in LSL (Fig. 2B). The overlap of 37 and 1 DEGs was observed being commonly regulated due to diet between the two strains at week 19 (Fig. 2A) and week 24 (Fig. 2B), respectively. Respective gene lists of the various comparisons of the dietary groups per sampling stage are provided in Table S2 (LBwk19P + vs. LBwk19P-), Table S3 (LSLwk19P + vs. LSLwk19P-), Table S4 (LBwk24P + vs. LBwk24P-), and Table S5 (LSLwk24P + vs. LSLwk24P-).

**Fig. 3:**
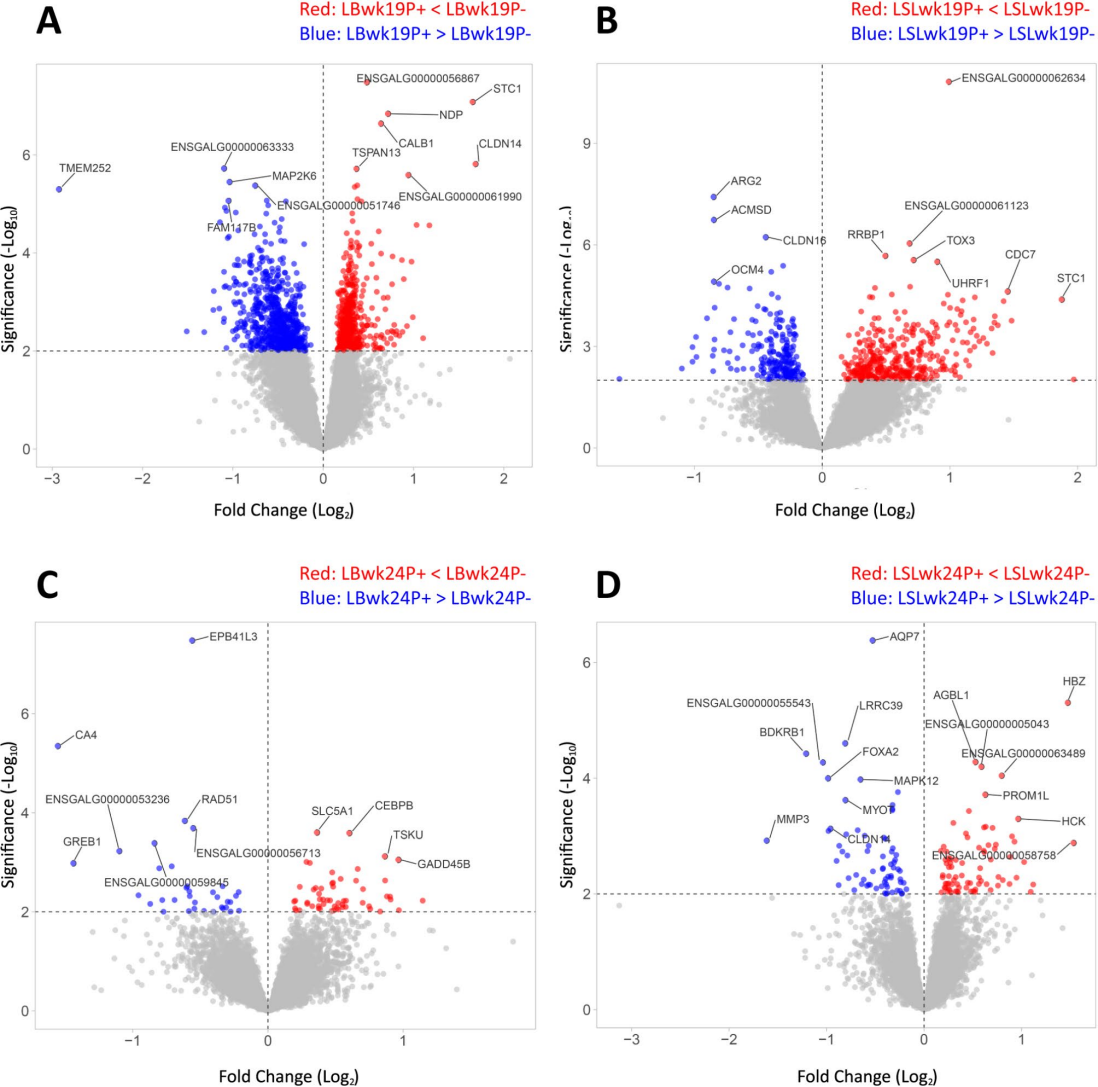
Volcano plots showing renal gene expression in comparison of a low P diet (P-) and a high P diet (P+) in LB (**A**) and LSL (**B**) laying hens at week 19, and in LB (C) and LSL (D) laying hens at week 24. Significant differentially expressed genes (DEGs) are depicted as red (P + < P-) and blue (P + > P-) dots. Gray dots remained unchanged due to dietary P supply

Volcano plots depicting gene expression data revealed distinct patterns in the LB and LSL strains at weeks 19 and 24 (Fig. 3). Before the onset of lay at week 19, the LB strain exhibited an upregulation (P + < P-) of *CALB1*, *CLDN14*, and *STC1*, among others, due to dietary P reduction, along with a downregulation (P + > P-) of *MAP2K6* (Fig. 3A). In the LSL strain, functionally interesting candidate genes such as *STC1* were upregulated (P + < P-), while *CLDN16* and *OCM4* were notably downregulated (P + > P-) (Fig. 3B). Following the onset of lay at week 24, the impact of the applied mineral P levels on renal gene expression was generally subtle. Regarding relevant functional candidate genes, in the LB strain, there was a significant upregulation (P + < P-) of *SLC5A1* and downregulation (P + > P-) of *CA4* and *GREB1* (Fig. 3C), whereas the LSL strain demonstrated significant downregulation (P + > P-) of *AQP7*,* MYOT*,* CLDN14* and *MAPK12* (Fig. 3D).

### Gene ontology and pathway enrichment analysis

KEGG pathway analysis did not provide pathways related to P homeostasis, however, an enrichment of pathways related to energy metabolism and the cell cycle was observed in both strains at week 19. Pathways pertinent to oxidative phosphorylation, phosphatidylinositol signaling system, and C-type lectin receptor signaling were highlighted in the LB strain. The LSL strain showed functional enrichment of genes associated with the metabolism of xenobiotics by cytochrome P450, cell cycle, pentose and glucuronate interconversions, and drug metabolism - cytochrome P450 (Table 1). No enriched pathways were found at week 24 for both LB and LSL laying hen strains.

**Table 1:** KEGG pathway analysis of differentially expressed genes (DEGs) with respect to strain (LB, LSL), dietary P supply (P+, P-), and stage (wk19, wk24)

Contrast of experimental groups	Pathway	Gene count	adjusted *P*-value
LBwk19P + vs. LBwk19P-	Oxidative phosphorylation	26	< 0.001
	Phosphatidylinositol signaling system	17	0.019
	C-type lectin receptor signaling pathway	16	0.038
LSLwk19P + vs. LSLwk19P-	Metabolism of xenobiotics by cytochrome P450	7	0.010
	Cell cycle	15	0.037
	Pentose and glucuronate interconversions	5	0.049
	Drug metabolism - cytochrome P450	6	0.047
LBwk24P + vs. LBwk24P-	-	-	-
LSLwk24P + vs. LSLwk24P-	-	-	-

### Diet-specific expression of target genes involved in P homeostasis

Based on the RNAseq dataset, a target gene approach was used to identify specific transcriptional responses linked with mineral utilization. Table 2 and Table S6 display the functional candidate genes which were upregulated (P + < P-) or downregulated (P + > P-) according to the RNAseq expression data in LB and LSL strains at weeks 19 and 24. At week 19, the genes *STC1*,* CALB1*,* CA2* and *CLDN14* were upregulated in both LB and LSL when comparing the different P diets (P + < P-). The gene encoding the P transporter *SLC34A1* was significantly upregulated, while *SLC20A2*, *SLC34A2* and other solute carriers such as *SLC2A13* were significantly downregulated in LB strain at week 19 (P + > P-). Significant upregulation of *SLC4A1*, *SLC8A1*, SLC41A2, *TRPV6* and *TRPM7* and downregulation of *CLDN16* was observed in LSL at week 19. *CLDN10*, *AQP11* and *SLC41A3* was upregulated, while *TJP1* and *ATP2B4* were downregulated in LB strain at week 19. The *CALCRL* gene linked to Ca control was significantly downregulated in LB at week 19.

After the onset of lay at week 24, the Ca exchanger encoding gene *SLC8A1* was downregulated in the LB strain while the P transporter gene *SLC20A1* was downregulated in the LSL strain when comparing the different P diets (P + > P-). In both strains, the aquaporin *AQP7* was significantly upregulated at week 24. In addition, *CA4* was downregulated in the LB strain. In the LSL strain at week 24, *CLDN14* and *STC1* were upregulated, with a downregulation observed in *ATP2B4.* Regarding vitamin D metabolism, *FGFR1* in LSL and *CYP24A1* in LB were significantly downregulated.

**Table 2:** Target genes with functions and their expression in LB and LSL laying hen strains following variable dietary P supply at week 19 and week 24. The negative log_2_-FoldChange values indicate lower mRNA abundances (P + > P-) and the positive log_2_-FoldChange values indicate higher mRNA abundances (P + < P-) in animals kept on low P diets (P-) compared to controls (P+)

			LB		LSL		
Gene ID	Gene Name	Expression (baseMean)	log_2_-Fold change	*P*-value	log_2_-Fold change	*P*-value	Functional category
*Week 19*							
AQP11	Aquaporin 11	871.7	0.40	0.009	0.05	0.701	water channel activity
ATP2B4	ATPase plasma membrane Ca2 + transporting 4	241.1	-0.38	0.027	0.03	0.875	calcium transport
CA2	Carbonic anhydrase 2	6114.9	0.24	0.008	0.38	0.009	hydration of carbon dioxide to form bicarbonate
CALB1	Calbindin 1	7142.8	0.64	< 0.001	1.00	< 0.001	calcium binding capability
CALCRL	Calcitonin Receptor Like Receptor	80.5	-0.33	0.031	0.13	0.398	calcium regulation
CLDN10	Claudin 10	4976.9	0.25	0.002	-0.21	0.110	passive transport
CLDN14	Claudin 14	38.9	1.68	< 0.001	1.38	< 0.001	passive transport
CLDN16	Claudin 16	370.4	0	0.995	-0.44	< 0.001	passive transport
SLC2A13	Solute carrier family 2 member 13	180.0	-0.51	0.015	-0.09	0.745	*myo*-inositol: proton symporter activity
SLC4A1	Solute carrier family 4 member 1	271.1	-0.14	0.357	0.26	0.029	chloride/bicarbonate exchanger
SLC8A1	Solute carrier family 8 Member 1	668.1	0.06	0.782	0.67	0.002	intracellular Ca transport
SLC20A2	Solute carrier family 20 member 2	1486.4	-0.28	0.031	-0.15	0.195	P transport
SLC34A1	Solute carrier family 34 member 1	41142.8	0.38	0.025	0.28	0.200	P transport
SLC34A2	Solute carrier family 34 member 2	11.8	-0.80	0.031	-0.18	0.573	P transport
SLC41A2	Solute carrier family 41 Member 2	111.2	-0.01	0.943	0.35	0.025	Mg transport
SLC41A3	Solute carrier family 41 Member 3	418.1	0.31	0.031	0.03	0.835	Mg transport
STC1	Stanniocalcin-1	57.2	1.65	< 0.001	1.87	< 0.001	Ca and P transport
TJP1	Tight junction protein 1	556.2	-0.33	0.020	0.26	0.181	passive transport
TRPM7	Transient receptor potential cation channel subfamily M member 7	280.8	-0.25	0.102	0.46	0.015	Ca and Mg transport
TRPV6	Transient receptor potential cation channel, subfamily V, member 6	279.0	-0.26	0.325	0.81	0.009	Ca transport
*Week 24*							
AQP7	Aquaporin 7	1184.3	0.40	0.003	0.53	< 0.001	water channel activity
ATP2B4	ATPase plasma membrane Ca2 + transporting 4	306.9	0.03	0.790	-0.25	0.030	calcium transport
CA4	Carbonic anhydrase 4	31.8	-1.56	< 0.001	-0.141	0.596	hydration of carbon dioxide to form bicarbonate
CLDN14	Claudin 14	39.0	0.03	0.932	0.96	0.001	passive transport
CYP24A1	Cytochrome P450 Family 24 Subfamily A Member 1	3127.6	-1.30	0.026	0.46	0.365	vitamin D elimination
FGFR1	Fibroblast Growth Factor receptor 1	1045.1	0.05	0.579	-0.22	0.039	P reabsorption
SLC8A1	Solute carrier family 8 Member 1	438.3	-0.42	0.012	-0.23	0.211	intracellular Ca transport
SLC20A1	Solute carrier family 20 member 1	678.3	-0.08	0.831	-0.84	0.017	P transport
STC1	Stanniocalcin-1	179.7	0.08	0.867	0.70	0.005	Ca and P transport

## Discussion

Prior to the onset of egg laying, a series of complex biological processes take place that are controlled by the endocrine system and comprise the gastrointestinal tract as well as tissues involved in post-absorptive metabolism such as kidney and liver. These processes regulate nutrient and mineral utilization based on developing needs in reproductive birds. The present study focused on animal-intrinsic effects of P homeostasis in the kidney and associated plasma metabolites, as the feed composition remained consistent at the two examined sampling stages to avoid confounding effects according to the interaction of diet × stage.

### Metabolic adaptation via plasma hormones and metabolites

The resulting plasma P reflected the dietary P intake in both strains at week 19, i.e. lowered P levels in P- diets, aligning with reports on reduced serum P values in P- fed hens at 25 weeks [36]. As the P- diets lacked mineral P and phytase supplements, the plasma P levels could be attributed to dietary NPP (1.3 g/kg) and phytate P in feed partially available due to mucosal and microbial enzyme activity [37]. Stage effects appeared to be significant both in LB and LSL with a decrease in plasma P levels from week 19 to 24 in accordance with previous studies [38]. The elevated plasma Ca values from week 19 to 24 are due to higher Ca intake following increased feed intake [27] and mobilization due to intensified Ca demand for eggshell synthesis [39]. The increase in ingested Ca led to a drop in plasma P levels as high Ca impairs the P absorption and phytate degradation by raising digesta pH [13] and forming Ca-phytate complexes [14]. In addition, these dynamics in blood Ca and P levels in matured laying hens are also a response to the significantly increased mineral turnover. Indeed, the medullary bone is involved in intermediate deposition of minerals and their rapid mineral mobilization during sexual maturation, which is important for the onset of eggshell production [40].

Through ALP activity, Ca mobilization from the bone and its deposition onto eggshell can be enhanced [41], thus the observed increase in ALP activity in LSL hens after onset of lay (week 24) may follow Ca needs. High levels of plasma Ca are known to inhibit the secretion of PTH from parathyroid glands [42], which might explain the decrease in PTH from week 19 to 24 in P + fed LSL hens. LSL hens fed P + diets could also reduce PTH secretion to ensure renal P reabsorption. The increase in triacylglycerides at week 24 corresponds to the involvement of hepatic metabolism for egg production. Since large amounts of the egg yolk proteins and lipids are synthesized in the liver and transported to the ovary during the laying period [43], triacylglyceride levels in blood increased about 10-fold after the onset of lay. The fluctuations in the plasma levels of albumin and Mg could be due to their deposition in the egg and thus the individual state of sexual maturity of the hens. The more pronounced effects of diet and sampling stage on plasma metabolites in LSL compared to LB hens might reflect a presumably higher degree of sexual maturity of LSL compared to LB.

Strain-specific differences of the analyzed plasma metabolites were pronounced for calcidiol, i.e. the storage form of vitamin D. This is consistent with previous reports where higher calcidiol levels were found in LB compared to LSL laying hens [11, 44]. Although no significant effects of dietary P supply and stage were observed for calcitriol levels, the numerical differences suggest established animal-intrinsic mechanisms acting on renal calcitriol synthesis. These endocrine adaptations may help to increase the intestinal mineral absorption following low P intake [45] and to meet the mineral requirements due to egg yolk deposition as well as eggshell calcification [46]. The variation in plasma calcitriol levels between hens, particularly at week 24, may reflect the individual status in sexual maturity and the associated adaptations of intestinal and post-absorptive tissues.

### Transcriptional profiling reveals metabolic interlinkages

In addition to variations observed at the plasma level, the difference in renal expression profiles between the two strains also indicates that the strains have adapted in a strain-specific manner, as has been previously suggested in relation to mineral homeostasis [5]. This difference in intrinsic mechanism driving mineral homeostasis corresponds to the genetic background of these strains [47]. Accordingly, the KEGG pathway enrichment analyses revealed pathways attributed to energy metabolism in LB hens and cell cycle in LSL hens at week 19. Likewise, pronounced strain differences in terms of immunity and intestinal capacity were described previously [26, 48]. The lack of enriched pathways in the kidney following varying mineral P supply at week 24 in both strains could be attributed to the considerable dynamics in Ca turnover at onset of egg laying which seems to outweigh the effects of dietary P reduction.

The expression pattern of the target genes found in our RNAseq data supported the role of the kidney in the regulation of P and Ca homeostasis in laying hens, as the adaptive capacity of kidney in mineral homeostasis was reported by previous studies [49]. *SLC34A1* encoding NPt2a was identified as the predominantly expressed Na/P co-transporter in the avian kidney [12]. The higher mRNA abundance of *SLC34A1* in LB at week 19 under low P diets is linked with enhanced P reabsorption in the kidney, as discussed previously for broiler chickens [50]. Similarly, a low P diet (0.15% vs. 0.41% vs. 0.82% available P) revealed an upregulated *SLC34A1* expression in kidney of laying hens as reported previously [32]. However, no effect on avian renal *SLC34A1* expression following a reduction in dietary NPP levels (0.14% vs. 0.32% NPP) was reported previously [51]. The annotated Na/P co-transporter *SLC34A2* was expressed at very low levels in avian kidney. LB hens showed a reduced *SLC34A2* mRNA abundance under P- diets as observed previously in growing broiler chickens [12]. Another Na/P co-transporter, *SLC20A2*, was downregulated (P + > P-) in LB at week 19 similar to findings in kidney of growing broiler chickens [12], while a higher expression was reported in fattening pigs [52] and rats [53] when subjected to a low P diet. Since the *SLC20A2* gene plays a housekeeping role in cellular P uptake by encoding PiT2 [54], its higher expression under P + diets could be linked to reduced cellular P efflux and prioritize available P for essential metabolic processes. The rate of P reabsorption to adjust plasma P levels may involve Na/P co-transport activity through hormonal control such as PTH and vitamin D [55], but may also be effectively regulated by *STC1*, which enhances renal P reabsorption and prevents hypercalcemia [56]. The considerable upregulation of *STC1* (P + < P-) observed in our study was similar to results in porcine kidney studying effects of low P diets [57]. The regulatory role of STC1 is considered a conserved mechanism, as also in fish stanniocalcin lowers Ca uptake by the gills and intestine and increases P reabsorption by the renal proximal tubules through an adenosine 3′,5′-cyclic monophosphate (cAMP)-dependent pathway, resulting in a decrease in Ca and increase in P levels [58].

In terms of passive mineral transport, claudin-14 (*CLDN14*) was significantly upregulated in P deficient groups in both strains at week 19 and only in LSL at week 24. The upregulation could be linked with maintaining Ca levels in the blood as *CLDN14* was reported to regulate the paracellular reabsorption of Ca and Mg in the thick ascending limb in humans [59]. The observed upregulation of claudin-10 (*CLDN10*) in the LB group at week 19 corresponds to its role in regulating Ca and Mg transport in the thick ascending limb of the kidney. This gene encodes two alternatively spliced isoforms of claudin-10 proteins in humans where claudin-10a functions as an anion channel, and claudin-10b acts as a cation channel [60]. Moreover, claudin-16 (*CLDN16)* was downregulated in LSL at week 19, while tight junction protein coding gene *TJP1* was downregulated in the LB group at week 19. *CLDN16* mediates Mg and Ca resorption through paracellular permeation in a porcine renal cell line [61]. Therefore, the renal expression profile provides evidence that both active and passive transport mechanisms were affected by the dietary mineral P level, although the specific gene functions in birds can so far only be derived from mammalian research. Interestingly, the varying mineral P level revealed an effect on aquaporin 7 (*AQP7*) and aquaporin 11 (*AQP11*), belonging to the transmembrane water and small solute channels family involved in water and glycerol transport. Although their role in Ca and P transport is still unknown, the higher expression of *AQP11* under P- diets in LB laying hens at week 19 is aligned with previous studies, where *AQP11* was significantly upregulated in the kidney of reproductive pigs due to a low P diet [62]. It is therefore conceivable that the dietary P intake and subsequent endogenous responses comprise secondary effects on the water balance.

The onset of egg production in laying hens necessitates a higher Ca turnover to meet the demands of eggshell formation. Calbindin-D28k, encoded by *CALB1*, plays a crucial role in regulating renal Ca and Mg reabsorption in the distal nephrons [63]. In the current study, *CALB1* was highly expressed in both strains of laying hens at week 19, with an upregulation observed in response to the P- diet. However, previous studies reported conflicting findings regarding the expression levels of *CALB1* and its encoded protein in the kidneys of laying hens subjected to varying dietary mineral levels at different stages [32, 33, 64]. Interestingly, while the ovulatory cycle did not significantly affect *CALB1* expression in the kidney, a significant response was observed in the shell gland [65]. In our study, *CALB1* expression levels indicate effectiveness of calcitriol-mediated renal mineral reabsorption, although calcitriol was not significantly but numerically increased by the applied P- diet.

The downregulation of *CYP24A1* due to P- diets in LB at week 24 suggests a reduction in renal 24-hydroxylation of calcitriol and thus reduced excretion via urine. This is consistent with the results from pigs fed low P diets [34], consequently ensuring adequate circulating levels of calcitriol, similar to the results described in laying hens [66]. In this context, downregulation of *CALCRL* in LB hens at week 19 may indicate calciotrophic effects to diminish the calcitonin-mediated inhibition of Ca reabsorption in the kidney, supporting a renal response to P- diets maintaining Ca homeostasis. However, the contribution of Ca regulation by calcitonin in birds remains unclear both in terms of efficacy and signal transduction [65]. In addition, systemic effects triggered by the phosphatonin FGF23 can be observed based on the renal transcription profile. The downregulation of fibroblast growth factor receptor 1 (*FGFR1*) in LSL at week 24 is consistent with a previous study, which revealed a reduced *FGFR1* expression following low P diets in laying hens [51]. Reduced FGF23 signaling ultimately decreases P excretion [67], as FGF23 negatively affects renal vitamin D biosynthesis by activating the catabolic enzyme CYP24A1 and suppresses renal P reabsorption by decreasing the expression of *SLC34A1* [68]. This might correspond to recent findings as LSL laying hens revealed significantly reduced P excretion compared to LB laying hens [27]. However, FGF23 also depends on its soluble receptor α-Klotho (KL), the renal expression of which remained unaltered due to P diets in accordance with earlier findings [51].

In order to maintain a physiological Ca: P ratio in the body compartments, the results indicate that both laying hen strains have adapted to the lack of additional mineral P sources by regulating Ca metabolism, as evidenced by changes in gene expression patterns in the kidney. The pronounced effect of the experimental factors on Ca metabolism is further illustrated by the variable expression of other key genes, including *TRPV6*, *SLC8A1* and *ATP2B4*. In fact, these genes are known to facilitate Ca transport whereas their role in renal Ca utilization is sparsely described in poultry [69]. Notably, the CaSR was constantly expressed in the kidney across all experimental groups, with no evidence of regulation due to dietary P reduction. In addition to DEGs involved in Ca metabolism, the divergent P diets also tackled genes encoding transcripts involved in Mg metabolism. In LSL hens at week 19, *TRPM7* was upregulated due to P- diet, which paralleled the numerically higher levels of plasma Mg. *TRPM7* regulates transcellular Mg reabsorption in the distal convoluted tubule of kidney [70]. Moreover, the upregulation of *SLC41A2* in LB and *SLC41A3* in LSL at week 19 indicates further interlinkages between P and Mg homeostasis as the SLC41 family is involved in Mg transport and indirectly regulates renal Mg reabsorption [71, 72].

The transcription profile showed that the *SLC4A1* abundance was higher in the P- group compared to the P + group in LSL at week 19, whereas the opposite was observed in reproductive pigs [62]. *SLC4A1* is an anion exchanger and mainly involved in intracellular bicarbonate (HCO₃⁻) and extracellular chloride (Cl⁻) exchange [73]. Moreover, an effect due to dietary P level was observed at the expression levels of carbonic anhydrases *CA2* and *CA4*. *CA2* was upregulated due to the P- diet in both strains at week 19. The Ca^2+^ and HCO_3_^−^ ions are continuously supplied to the eggshell gland via transepithelial transport [74] to form calcium carbonate (CaCO₃). The upregulation of *CA2* was in line with previous findings obtaining a 4-fold increased expression of *CA2* in shell gland of laying hens during oviposition [65]. *CA2* was also identified in quail renal tubules [75] as a cytosolic coenzyme for bicarbonate reabsorption in the avian kidney. *CA2* is considered the major cytoplasmic isoform of carbonic anhydrase in the nephron, while a minor activity is thought to be delivered by *CA4*. *CA4*, also described for its activity in bicarbonate reabsorption and transport mechanisms in rabbit kidney cells [76], was not expressed at week 19, however, the transcript was expressed at a low rate at week 24 and was significantly downregulated in LB hens due to the P- diet. There is evidence that canine carbonic anhydrases are involved in the regulation of P excretion through an unknown mechanism, likely affecting acid-base balance in the kidneys [77].

## Conclusions

Both LB and LSL strains subjected to a diet without mineral P exhibited strain-specific endogenous adaptation to maintain P homeostasis via renal gene expression and endocrine profiles. The lack of dietary mineral P caused endogenous responses prior to the onset of laying in week 19, which, however, declined with increasing maturity of the hens. Associated metabolic adaptation showed particular significance for Ca homeostasis and triglycerides. Differential expression analysis underlines the important role of the renal functions for mineral homeostasis, i.e. passive/active mineral transport, Ca transport, and vitamin D elimination. Transcriptional profiling revealed new P-dependent targets such as carbonic anhydrase 2 (*CA2*) in both laying hen strains, which might offer new insights into metabolic interlinkages of mineral P depletion as well as trade-offs for animal health and performance.

## Electronic supplementary material

Below is the link to the electronic supplementary material.

Supplementary Material 1

## Data Availability

Data from RNA-sequencing are available from the EMBL-EBI database (www.ebi.ac.uk/arrayexpress) via the accession number E-MTAB-14436.

## Abbreviations

ALP: Alkaline Phosphatase Activity
Ca: Calcium
CaCO₃: Calcium Carbonate
ELISA: Enzyme-Linked Immunosorbent Assays
FGF23: Fibroblast Growth Factor 23
LB: Lohmann Brown
LSL: Lohmann Selected Leghorn
Mg: Magnesium
Na/P: Sodium/Phosphate
NPP: Non-Phytate Phosphorus
P: Phosphorus
P+: High Phosphorus Diet
P-: Low Phosphorus Diet
PCR: Polymerase Chain Reaction
PTH: parathyroid hormone
RIN: RNA Integrity Numbers
1,25-(OH)2D3: Calcitriol (Active Form of Vitamin D3)
25(OH)D3: Calcidiol (Storage Form of Vitamin D3)
25(OH)D: 25-Hydroxyvitamin D
1,25-(OH)₂D: 1,25-Dihydroxyvitamin D
